# Supplementary material for: Clinical value of questionnaires & physical tests for patellofemoral pain: Validity, reliability and predictive capacity
Source: PLoS One. 2024 Apr 17;19(4):e0302215. doi: 10.1371/journal.pone.0302215 (PMC11023591; doi:10.1371/journal.pone.0302215)
Supplement: Attachment — Submitted filename: Response to Reviewers.docx [file pone.0302215.s002.docx]

Dear Dr. Espuny-Ruiz,

Thank you for submitting your manuscript to PLOS ONE. After careful consideration, we feel that it has merit but does not fully meet PLOS ONE’s publication criteria as it currently stands. Therefore, we invite you to submit a revised version of the manuscript that addresses the points raised during the review process.

Please submit your revised manuscript by April 04 2024 11:59PM. If you will need more time than this to complete your revisions, please reply to this message or contact the journal office at [plosone@plos.org](mailto:plosone@plos.org). When you're ready to submit your revision, log on to https://www.editorialmanager.com/pone/ and select the 'Submissions Needing Revision' folder to locate your manuscript file.

Dear Mehrnaz Kajbafvala, Ph.D, Academic Editor,

We appreciate your response to our manuscript and the effort you have made to improve it. We have carefully read all your comments and those of the reviewers, responding to each of them point by point.

Thanks to your expert suggestions we have revised the manuscript, clarified some aspects of the text, made grammatical modifications, corrected some mistakes, attached the required documents (database as a supplementary file and informed consent; informed assent of the minor and ethics committee approval for the journal), etc. All modifications to the Main Text have been made with track changes.

We believe that the current manuscript is more complete and rigorous than the original. We hope we have met the expectations of your prestigious journal.

Our responses are shown point by point below. We remain at your disposal for any further suggestions.

Best regards,

Fernando Espuny-Ruiz, MSc (corresponding author 1)

Gema Chamorro-Moriana, PhD (corresponding author 2)

Please include the following items when submitting your revised manuscript:

- A rebuttal letter that responds to each point raised by the academic editor and reviewer(s). You should upload this letter as a separate file labeled 'Response to Reviewers'.

We have sent the requested document with point-by-point answers, “Response to Reviewers”.

- A marked-up copy of your manuscript that highlights changes made to the original version. You should upload this as a separate file labeled 'Revised Manuscript with Track Changes'.

We have sent the requested document with point-by-point answers, “Revised Manuscript with Track Changes”

- An unmarked version of your revised paper without tracked changes. You should upload this as a separate file labeled 'Manuscript'.

We have sent the requested document with point-by-point answers, “Manuscript”

If you would like to make changes to your financial disclosure, please include your updated statement in your cover letter. Guidelines for resubmitting your figure files are available below the reviewer comments at the end of this letter.

Not applicable.

If applicable, we recommend that you deposit your laboratory protocols in protocols.io to enhance the reproducibility of your results. Protocols.io assigns your protocol its own identifier (DOI) so that it can be cited independently in the future. For instructions see: <https://journals.plos.org/plosone/s/submission-guidelines#loc-laboratory-protocols>. Additionally, PLOS ONE offers an option for publishing peer-reviewed Lab Protocol articles, which describe protocols hosted on protocols.io. Read more information on sharing protocols at <https://plos.org/protocols?utm_medium=editorial-email&utm_source=authorletters&utm_campaign=protocols>.

Not applicable.

We look forward to receiving your revised manuscript.

Kind regards,

Mehrnaz Kajbafvala, Ph.D
Academic Editor
PLOS ONE

Journal Requirements:

When submitting your revision, we need you to address these additional requirements.

1. Please ensure that your manuscript meets PLOS ONE's style requirements, including those for file naming. The PLOS ONE style templates can be found at 
https://journals.plos.org/plosone/s/file?id=wjVg/PLOSOne_formatting_sample_main_body.pdf and 
<https://journals.plos.org/plosone/s/file?id=ba62/PLOSOne_formatting_sample_title_authors_affiliations.pdf>.

2. Please provide additional details regarding participant consent. In the ethics statement in the Methods and online submission information, please ensure that you have specified what type you obtained (for instance, written or verbal, and if verbal, how it was documented and witnessed). If your study included minors, state whether you obtained consent from parents or guardians. If the need for consent was waived by the ethics committee, please include this information.

Thank you for the information. The authors have expanded the information *(italics),* as well as attached three files for the journal with the authorization of the ethics committee (File: Ethics Committee Approval), the informed consent (File: Informed Consent Spanish & English) and the informed assent of the minor (File: Informed Assent U-18 Spanish & English). The text (Manuscript - lines 244-256; Revised Manuscript with Track Changes - lines 250-262) is as follows: “This research was conducted in accordance with the Declaration of Helsinki and approved on 3 December 2021 by the Research Ethics Committee of the Virgen Macarena-Virgen del Rocío Hospitals of the Andalusian Public Health System (C.I.0162-N-21). *Before starting the fieldwork (request for personal data and functional assessment), all participants received a written information sheet about the study together with the informed consent to sign. They were able to ask questions and solve any doubts about these documents with the researchers before signing the consent, which did not prevent the subject from leaving the study if he/she wanted to.* All patients gave written informed consent after reading the user information sheet. *For those subjects under 18 (inclusion criterion: minimum 16 years), the consent signatures of the parent or legal guardian and the informed assent of the minor, i.e. in age-appropriate language, were expressly required. All the above was included in the application submitted to the Ethics Committee for approval.”*

3. We note that your Data Availability Statement is currently as follows: All relevant data are within the manuscript and its Supporting Information files.

Please confirm at this time whether or not your submission contains all raw data required to replicate the results of your study. Authors must share the “minimal data set” for their submission. PLOS defines the minimal data set to consist of the data required to replicate all study findings reported in the article, as well as related metadata and methods (<https://journals.plos.org/plosone/s/data-availability#loc-minimal-data-set-definition>).

For example, authors should submit the following data:

- The values behind the means, standard deviations and other measures reported;
- The values used to build graphs;
- The points extracted from images for analysis.

Authors do not need to submit their entire data set if only a portion of the data was used in the reported study.

If your submission does not contain these data, please either upload them as Supporting Information files or deposit them to a stable, public repository and provide us with the relevant URLs, DOIs, or accession numbers. For a list of recommended repositories, please see <https://journals.plos.org/plosone/s/recommended-repositories>.

Thank you for the information. The authors have attached all the necessary data in a supplementary file (S1 Data) for the reproduction of the study (Sex, age, height, body mass index, bilateral or unilateral impairment, affected knee, dominant limb, time with pain, Q-angle, chondromalacia patellae degree according to magnetic resonance imaging, KOOS-PF, KPS and VISA-P points in both measurement moments, physical tests results and specific items of each questionnaire linked to the squat test points).

If there are ethical or legal restrictions on sharing a de-identified data set, please explain them in detail (e.g., data contain potentially sensitive information, data are owned by a third-party organization, etc.) and who has imposed them (e.g., an ethics committee). Please also provide contact information for a data access committee, ethics committee, or other institutional body to which data requests may be sent. If data are owned by a third party, please indicate how others may request data access.

Not applicable.

4. PLOS requires an ORCID iD for the corresponding author in Editorial Manager on papers submitted after December 6th, 2016. Please ensure that you have an ORCID iD and that it is validated in Editorial Manager. To do this, go to ‘Update my Information’ (in the upper left-hand corner of the main menu), and click on the Fetch/Validate link next to the ORCID field. This will take you to the ORCID site and allow you to create a new iD or authenticate a pre-existing iD in Editorial Manager. Please see the following video for instructions on linking an ORCID iD to your Editorial Manager account: <https://www.youtube.com/watch?v=_xcclfuvtxQ>.

Thank you for the information. The authors have checked all the information related to ORCID and have updated it.

5. Please review your reference list to ensure that it is complete and correct. If you have cited papers that have been retracted, please include the rationale for doing so in the manuscript text, or remove these references and replace them with relevant current references. Any changes to the reference list should be mentioned in the rebuttal letter that accompanies your revised manuscript. If you need to cite a retracted article, indicate the article’s retracted status in the References list and also include a citation and full reference for the retraction notice.

Thank you for your suggestion. We have checked all the references point by point. Everything is correct. In addition, following the recommendations of reviewer 1, new citations have been added in the introduction section and even two new references has been added (current references 19 and 42). The citation numbering and reference identifiers have been modified across the manuscript.


[Note: HTML markup is below. Please do not edit.]

Reviewers' comments:

Reviewer's Responses to Questions

**Comments to the Author**

1. Is the manuscript technically sound, and do the data support the conclusions?

The manuscript must describe a technically sound piece of scientific research with data that supports the conclusions. Experiments must have been conducted rigorously, with appropriate controls, replication, and sample sizes. The conclusions must be drawn appropriately based on the data presented.

Reviewer #1: Yes

Reviewer #2: Yes

2. Has the statistical analysis been performed appropriately and rigorously?

Reviewer #1: Yes

Reviewer #2: Yes

3. Have the authors made all data underlying the findings in their manuscript fully available?

The [PLOS Data policy](http://www.plosone.org/static/policies.action#sharing) requires authors to make all data underlying the findings described in their manuscript fully available without restriction, with rare exception (please refer to the Data Availability Statement in the manuscript PDF file). The data should be provided as part of the manuscript or its supporting information, or deposited to a public repository. For example, in addition to summary statistics, the data points behind means, medians and variance measures should be available. If there are restrictions on publicly sharing data—e.g. participant privacy or use of data from a third party—those must be specified.

Reviewer #1: Yes

Reviewer #2: Yes

4. Is the manuscript presented in an intelligible fashion and written in standard English?

PLOS ONE does not copyedit accepted manuscripts, so the language in submitted articles must be clear, correct, and unambiguous. Any typographical or grammatical errors should be corrected at revision, so please note any specific errors here.

Reviewer #1: Yes

Reviewer #2: Yes

5. Review Comments to the Author

Please use the space provided to explain your answers to the questions above. You may also include additional comments for the author, including concerns about dual publication, research ethics, or publication ethics. (Please upload your review as an attachment if it exceeds 20,000 characters)

Reviewer #1: This study was aimed to determine the most appropriate method of functional assessment for "patellofemoral pain" (PFP)/“chondromalacia patella” for its diagnostic value, (validity, reliability, sensitivity, specificity, predictive value and clinical applicability); to outline initial interpretations of the questionnaires and their appropriateness, through the cutoff points determined in their scores based on physical test and Magnetic Resonance Imaging (MRI); to establish which methods should be used in conjunction with each other to obtain clinical diagnoses that are robust effective and efficient. The study findings indicated that KOOS-PF, KPS and VISA-P demonstrated their diagnostic value in PFP/chondromalacia (validity, reliability, sensitivity, specificity, predictive value and clinical applicability). KOOS-PF was the most versatile, and the most appropriate in mild cases and for early detection and prevention. Squat was the best due to its reliability and clinical relationship with the questionnaires, which predicted it correctly. The methods discussed should be applied in combination. Overall, the study is interesting, however there are several clarifications needed.

Comment#1
Title is not appropriate. Please state title more accurately, scientifically and concisely.

Thank you for your comment. The title has been modified following your suggestion: “Clinical value of questionnaires & physical tests for patellofemoral pain: validity, reliability and predictive capacity.”

Comment#2
Please edit Keywords. Keywords should be the reflective of the research performed. In addition to predictive capacity, intra- and inter-observer reliability and clinical usefulness were assessed in this study.

Thank you for your suggestion. The authors have modified the Keywords following your comment and trying as much as possible to use MeSH terms: Diagnosis, Functional assessment, Validation study, Reliability, Predictive value of tests, Patellofemoral Pain, Chondromalacia Patellae.

We remain at your disposal if you consider that we should make any other changes.

Comment#3
Introduction. Please insert reference/references in different parts of Introduction section for supporting the sentences stated.

Thank you for your comment. We have revised the introduction, detecting that some cites are missing. We have added new citations to the existing references in the manuscript throughout the section (as this was done with a reference manager, the new citations are not reflected in the track changes), as well as including the bibliographic reference of two new studies (currently references 19 and 42). Both the numbering of the citations and the number assigned to each reference have been modified throughout the Manuscript.

Comment#4
Methods. How did you assess the preventive capacity of questionnaires & physical tests?

Thank you for your question. The study evaluated the appropriateness of the assessment methods from different perspectives. Among other factors, it was assessed which methods were most appropriate in early and more advanced stages. For example, the KOOS-PF scale is the most appropriate in mild cases and the KPS in more advanced stages, where there is a greater likelihood of structural cartilage damage, chronic pain and long-term sequelae. This study advocates early assessment, which in the case of patellofemoral pain/chondromalacia patellae would allow preventive treatment of such structural damage and chronicity. Treatment could target both the symptomatology (pain, stiffness, functional limitation, etc.) and the cause of the problem (dynamic knee valgus, muscle imbalance, foot or hip dysfunction, etc.). Therefore, knowing which methods are most sensitive to mild symptomatology would help to prevent the development of the pathology.

This idea is developed throughout the manuscript, which is included in the Conclusions section (Manuscript - lines 722-725; Revised Manuscript with Track Changes - lines 728-731): “…*Although the questionnaires analysed were found to be clinically feasible, KOOS-PF appeared to be the most versatile of the three, and, in general, the most appropriate in mild cases and for early detection and prevention. KPS is the most advisable in severe cases...”*.

Comment#5
Please state sample size calculation.

Thank you for the correction. We have added data about the sample size calculation, as well as the associated references, in the “*Statistical analysis*” subsection of the materials and methods section (Manuscript - lines 190-197; Revised Manuscript with Track Changes - lines 196-203): “*Sample size calculation was based on a related study that examined the combination of history elements and physical tests for PFP[25]. The following conditions were used: KOOS-PF mean standard deviations (s), 20.22; confidence interval of 95% and error (E), 4. The following formula used was:*

$$n=\frac{z_{\frac{\alpha}{2}\cdot}^{2}s^{2}}{E^{2}}$$

*The sample size obtained was 99 affected knees. Our sample included a few more subjects following COSMIN recommendations[42] (optimum ≥ 100) and anticipating possible dropouts.”*

Comment#6
Please state study limitations.

Thank you for your comment. We realized that we needed to clarify in the Manuscript the two limitations presented. One of them is associated with the exclusion of specific and validated questionnaires, but not adapted to Spanish (Manuscript - lines 692-695; Revised Manuscript with Track Changes - lines 698-701); and the second one is related to the size of the subsample used for the relationships among questionnaires and chondromalacia patellae degree according to MRI (Manuscript - lines 700-701; Revised Manuscript with Track Changes - lines 706-708). Both limitations will lead to prospective studies presented (Manuscript - lines 696-697, 702-703; Revised Manuscript with Track Changes - lines 702-703, 708-709).

The authors of this study sincerely thank you for your efforts to improve our Manuscript.

Best regards,

Reviewer #2: Thank you for providing us with the opportunity to review this manuscript. Overall, the topic is a nice job with some minor concerns, which I will outline below.

Line 52. What do you mean?

Thanks to your question, we have modified the sentence “*…functional assessment tools…, should be applied in combination*”, for better understanding. Both in the conclusions of the abstract and the main text. It reads as follows: “*…should be applied by combining them with each other*” (Manuscript - lines 50 and 742-743; Revised Manuscript with Track Changes - lines 53 and 748-749).

This same idea appears in the discussion section (Manuscript - lines 650-652; Revised Manuscript with Track Changes - lines 656-658) prior justification.

For example, if one functional assessment method better predicts early symptoms and another better predicts more advanced damage; or one is more suitable for those who are physically active and another for more sedentary people; we should use both methods to reach a more accurate diagnosis or even confirm the diagnosis. In addition, it is sometimes highly recommended, as stated in the conclusions, to combine functional assessment methods with some complementary tests (MRI or X-Ray).

Line 83-85. Please rewrite these sentences.

Thank you for your comment. The authors have rewritten the sentence following your instructions. We have added a comma after “*high grades”* to clarify the meaning of the sentence (Manuscript - lines 83-85; Revised Manuscript with Track Changes - lines 88-90). It reads as follows: “*Magnetic Resonance Imaging (MRI), which appears to be accurate in high grades[22], and arthroscopies; show low correlation between degrees and symptoms[22].”*

Line 93. “so non-specific questionnaires, such as the Fulkerson Knee Instability Scale (FKIS)[26], are also.. “What do you mean?

Thank you for your question. This sentence refers to the fact that, as there are few specific questionnaires to assess PFP (for instance, Kujala Patellofemoral Score), some researchers or clinicians have to use other non-specific questionnaires to assess the same pathology. This is the case of the Fulkerson Knee Instability Scale. There are various reasons for this, for example: the specific questionnaires do not have the necessary cross-cultural adaptation, they are not accessible to the practitioner or even unknown to him/her, etc.

Thanks to your comment, we have modified the original text to make it clearer (Manuscript - lines 92-95; Revised Manuscript with Track Changes - lines 97-101).

Original sentence: “*Few questionnaires are validated and created specifically for these patellofemoral conditions, i.e. Kujala Patellofemoral Score (KPS)[25], so non-specific questionnaires, such as the Fulkerson Knee Instability Scale (FKIS)[26], are also frequently used[19,20,27]”*

Modified sentences: “*Few questionnaires are validated and created specifically for these patellofemoral conditions, i.e. Kujala Patellofemoral Score (KPS)[26]. Since cross-cultural language adaptations are not always available or they are not accessible to researchers and clinicians, among others, non-specific questionnaires are also frequently used to assess PFP[20,21,28]. This is the case of Fulkerson Knee Instability Scale (FKIS)[27].”*

Line 129. Please clearly explain PFP diagnosis in your study participants. Did having musculoskeletal disorders in other joints as an exclusion criteria? Please explain exclusion criteria in detail.

Thank you for your questions and comments. We have clarified the text (Manuscript - lines 130-142; Revised Manuscript with Track Changes - lines 136-148) about the diagnosis of PFP in the inclusion criteria paragraph and expanded the exclusion criteria based on this clarification and musculoskeletal disorders of other joints. The amended criteria are as follows:

*“… Inclusion criteria were: subjects with symptomatic PFP with or without cartilage damage* *(note: by PFP we mean the patellofemoral joint conflict that produces friction between the facet joints due to non-traumatic causes such as knee valgus, muscle imbalance between the vastus medialis and vastus lateralis, etc., as shown in the introduction section)… Exclusion criteria were:…; PFP symptomatology common to other affected knee dysfunction* *such as femoral condyle or patella fractures affecting the facet joints, knee prosthesis, etc.; disorders of other joints such as hip, ankle or femorotibial joint, which prevent the performance of physical tests or bias the test results.”*

Line 143. “KPS” is “KOOS-PF”?

We have reviewed the text and it is correct. “KPS” refers to “Kujala Patellofemoral Score”, mentioned for the first time in line 92/97 (Maniscript/Revised Manuscript with Track Changes) of the introduction section, and KOOS-PF refers to “Knee injury and Osteoarthritis Outcome Score for Patellofemoral pain and osteoarthritis”.

Thank you for your question.

Line 145-146. Please edit this sentence grammatically.

Thank you for your recommendation. The authors have modified the sentence (Manuscript - lines 153-155; Revised Manuscript with Track Changes - lines 159-161) following your suggestion: “*Victorian Institute of Sports Assessment for Patellar tendons questionnaire (VISA-P), which is applied in PFP[29], although it was designed specifically for patellar tendinopathy[38]*”.

Line 168. Please explain words before abbreviating “FE-CR, FE-CR)

The acronyms belong to the names and surnames of the researchers who have carried out the tasks indicated before the acronyms, which, in this case, coincide with two of the co-authors of this study. For this reason, the authors have decided to use only the acronyms.

We remain at your disposal should you consider it appropriate to make a modification.

Please explain CP grad using MRI assessment.

Thanks to your comment we have detected a flaw in our manuscript. Thus, we have added information about the degrees of Chondromalacia Patella using Magnetic Resonance Imaging in the Materials and Methods section (Manuscript - lines 167-172; Revised Manuscript with Track Changes - lines 173-178). It reads as follows: “*MRI has been used in this study to establish cut-off points in the questionnaire scores according to the CP degrees it determines. The degrees[10] are: I, cartilage softening and swelling; II, fissures <0.5 inches in diameter not reaching the subchondral bone; III, fissures >0.5 inches reaching the subchondral bone; and IV, erosion that exposes subchondral bone.”*

The authors of this study are sincerely grateful for your efforts to enhance our manuscript.

Best regards,
